# Editorial Notes

**Published:** 1920-06

**Authors:** 


### Editorial Notes.

**Amalgamation of Medical Charities.**

Now that the provisional scheme for the amalgamation of the Bristol Hospitals has been considered by the Governors of the charities concerned, it need no longer be regarded as *sub judice*. The views of the Governors of the various charities have been expressed, with the result that the scheme set forward has not met with unanimous acceptance.

The attitude of the governing bodies towards the principle of co-operation or amalgamation is vastly more important than their attitude towards any provisional scheme for giving effect to amalgamation. It would have been wiser, perhaps, had the provisional scheme of the Organising Committee been submitted as an interim report to the bodies which had sent their representatives to the Organising Committee. Objections might have been weighed, and modifications made which would have averted the possibility that a rejection of the provisional scheme would be interpreted as a refusal to consider amalgamation or co-operation on any basis. Without sacrificing essential principles, it is permissible to suggest improvements in a scheme which goes so far into detail as this one.

Whilst the Governors of the Bristol Royal Infirmary accepted the scheme in the sense of a provisional draft, and contented themselves with indicating amendments which they would press at the proper time and place, the Children's Hospital gave a conditional approval. The Eye Hospital, the Bristol Dispensary and the Orthopædic Hospital expressed a readiness to fall in with the scheme. The Eye Dispensary and the Cossham Hospital preferred to remain outside for the present.

**Attitude of the General Hospital.**

The Committee of the General Hospital adopted a somewhat different attitude, and were supported by their general Board of Governors. They frankly rejected, not the principle of co-operation, nor that of amalgamation, but the provisional scheme by which it was proposed to effect the amalgamation. The grounds for their action deserve close examination, and in effect we conceive the standpoint of the General Hospital Committee to be this: When a hospital presents a splendid record in its business management, in its professional skill and scientific equipment, in a word in all that goes to provide devoted care for the sick, is it to be expected that the Governors will immediately and irrevocably surrender their trust?

**Spirit of Voluntary Hospitals.**

Voluntary hospitals, while serving a great national need, are a combination of business and philanthropy, into which sentiment cannot and should not be denied an entrance. No one wants to check the charitable and kindly impulse which brings subscriptions to provide hospital accommodation for those who are needy and sick, needy it may often be because they are sick. No one wishes to deny to such sentiment the means of gratifying the impulse, nor to exclude such subscribers from some share in managing the institution they support.

At the same time, a new and praiseworthy spirit of mutual help and support has awakened in the ranks of organised labour. The members of Trade Unions and Friendly Societies contribute during health to the maintenance of hospitals to which they may have occasion to resort. They too claim a share in the management. The medical staffs contribute to the common upkeep their gratuitous services. All alike are actuated, we think, by the same motive, which is in a sense altruistic and in a sense self-preservative: even on the lowest ground it is the herd-instinct working to protect the society in which we live. The objections raised to the provisional scheme cannot be lightly brushed aside. They must be met by adjustment of the scheme.

**Fusion or Partnership.**

Essentially the question turns on whether *amalgamation* is to involve immediate *fusion*, or whether close co-ordination and unification may, at the first offset, be experimental and in the nature of a *partnership* and capable of dissolution. It is impossible not to sympathise with the fears of Governors lest their institutions should be theirs no longer, it is impossible too not to share their fears lest the Central Council should become too bureaucratic in its constitution and methods. To ensure against the latter risk we can conceive no better safeguard than that the institutions entering into amalgamation should retain sufficient individuality to withdraw from the combine if it falls short in its usefulness.

We have not commented on the proposed recommendation that the University should be directly represented on the Central Council, because although we believe that close union between hospitals and University is mutually advantageous, the invitation to the University to co-operate can only be given by the combining hospitals if and when they decide to amalgamate.

Unquestionably the time has come for some measure of amalgamation and co-ordination of the hospital charities in Bristol. But in a matter where there is so large a basis of agreement uncompromising zeal may prove a hindrance to the cause it hopes to promote.

